# Exploring facial expressions and action unit domains for Parkinson detection

**DOI:** 10.1371/journal.pone.0281248

**Published:** 2023-02-02

**Authors:** Luis F. Gomez, Aythami Morales, Julian Fierrez, Juan Rafael Orozco-Arroyave

**Affiliations:** 1 Faculty of Engineering, Universidad de Antioquia, Medellín, Antioquia, Colombia; 2 School of Engineering, Universidad Autónoma de Madrid, Madrid, Madrid, España; 3 Pattern Recognition Lab, Friedrich-Alexander-Univertität Erlangen-Nürnberg, Erlangen, Germany; Politechnika Slaska, POLAND

## Abstract

**Background and objective:**

Patients suffering from Parkinson’s disease (PD) present a reduction in facial movements called hypomimia. In this work, we propose to use machine learning facial expression analysis from face images based on action unit domains to improve PD detection. We propose different domain adaptation techniques to exploit the latest advances in automatic face analysis and face action unit detection.

**Methods:**

Three different approaches are explored to model facial expressions of PD patients: (i) face analysis using single frame images and also using sequences of images, (ii) transfer learning from face analysis to action units recognition, and (iii) triplet-loss functions to improve the automatic classification between patients and healthy subjects.

**Results:**

Real face images from PD patients show that it is possible to properly model elicited facial expressions using image sequences (neutral, onset-transition, apex, offset-transition, and neutral) with accuracy improvements of up to 5.5% (from 72.9% to 78.4%) with respect to single-image PD detection. We also show that our proposed action unit domain adaptation provides improvements of up to 8.9% (from 78.4% to 87.3%) with respect to face analysis. Finally, we also show that triplet-loss functions provide improvements of up to 3.6% (from 78.8% to 82.4%) with respect to action unit domain adaptation applied upon models created from scratch. The code of the experiments is available at https://github.com/luisf-gomez/Explorer-FE-AU-in-PD.

**Conclusions:**

Domain adaptation via transfer learning methods seem to be a promising strategy to model hypomimia in PD patients. Considering the good results and also the fact that only up to five images per participant are considered in each sequence, we believe that this work is a step forward in the development of inexpensive computational systems suitable to model and quantify problems of PD patients in their facial expressions.

## Introduction

Parkinson’s Disease (PD) is a neurological disorder characterized by motor and non-motor impairments that affects between 1 and 2 percent of people over 65 years old [1]. Motor deficits include bradykinesia, rigidity, postural instability, tremor, and dysarthria; and non-motor deficits include depression, anxiety, sleep disorders, and slowing of thought. Besides the extensive list of symptoms, most patients with PD exhibit also difficulties to express emotions or specific expressions on their faces. Possible signs of those abnormalities include less range of facial muscle movement, wider opening of eyes, half-open mouth, and slower blinking. All of these phenomena in their facial expression are grouped in the literature and called hypomimia [2], which is the result of motor impairments at the facial muscles level. It is typically not noticed in early stages of PD, but once there is a significant deterioration, orofacial movements are highly reduced which can result in expressionless faces, with a very limited capability to smile, to express other emotions or feelings like happiness, sadness, anger, fear, disgust, and surprise [3]. The main effect of these impairments is in difficulties with non-verbal communication which also produces social isolation in a mid to long term.

Clinical evaluation of PD patients is mainly performed by expert neurologists according to the Movement Disorder Society—Unified Parkinson’s Disease Rating Scale (MDS-UPDRS) [4]. This scale is the global standard for the clinical evaluation of PD patients and it considers both motor and non-motor symptoms. Items of the MDS-UPDRS scale range between 0 and 4, where 0 means completely healthy and 4 means completely impaired. Section III in MDS-UPDRS has a maximum value of 132 and covers motor examination including facial expression in one item. According to the guidelines given by the Movement Disorder Society, the five levels of the item where hypomimia is evaluated can be used to assess facial expressions in PD patients [4]. The following list indicates the correspondence between possible values of the item and their meaning in terms of facial expression evaluation:

0. Normal: Normal facial expression.
1. Slight: Minimal masked facies manifested only by decreased frequency of blinking.
2. Mild: In addition to decreased eye-blink frequency, masked facies present in the lower face as well, namely fewer movements around the mouth, such as less spontaneous smiling, but lips not parted.
3. Moderate: Masked facies with lips parted some of the time when the mouth is at rest.
4. Severe: Masked facies with lips parted most of the time when the mouth is at rest.

Neurological evaluation highly depends on the clinician’s expertise, which causes variability and possible bias in the rating procedure. Therefore, the development of computerized systems to objectively support the evaluation of the disease progression is now growing in importance. There are several contributions in the state of the art where computerized systems are introduced to evaluate different aspects of Parkinson’s patients including speech [5, 6], gait [7, 8], handwriting [9–12], hands movement [13], and facial expression [14]. Among all, facial expression and hypomimia seem to be the least covered. Facial Expression Recognition (FER) refers to the evaluation of the capability of PD patients to effectively recognize different expressions or emotions when watching at faces. Facial Expressivity Evaluation (FEE) refers to the capability of the patient to produce different facial expressions or emotions. Both aspects have a very important role in social interaction and non-verbal communication. The first one has been studied for several decades mainly by psychologists in different works and the main findings are summarized in a relatively recent study [15]. On the other hand, FEE has become a popular field among engineers and computer scientists, which opens space for research in different applications related to affective computing.

During the past two decades, the affective computing community has made great advances in developing novel technologies to model facial expressions and emotional information [16–18]. One of the goals of affective technologies is to create computational models with the ability to recognize, interpret, and process human emotions, making human-computer interaction more useful. Sentiment analysis and affective computing have been continuously studied since the 20th century, helping in the development of computer vision systems [19–21], in the creation of entertainment [22], and in the development of systems to aid different areas of medicine including neurology [23–25].

Our work is focused on the study of FEE in PD patients. The main aim is to consider videos collected from patients to evaluate their capability to elicit specific facial expressions and to compare such a capability with respect to healthy subjects using recent advances in Action Unit domains. This work presents three different approaches: (i) the face analysis domain which is based on single images and image sequences extracted from the participants’ videos, (ii) the action unit domain which is created by applying transfer learning from the face analysis domain, and (iii) a specific analysis domain, focused on information from PD patients, that results from using the triplet loss function to improve the classification between PD patients and healthy subjects.

The rest of the paper is organized as follows: Related Works provides an overview about the literature on FEE. Contributions of this Work describes the contributions of this work in the topic of hypomimia modeling in Parkinson’s disease. Materials and Methods presents the experimental framework, including the description of the datasets and the methods. Experiments and Results summarizes the experiments and results. Finally, the discussion, conclusions and future work are drawn in Discussion and Conclusion.

### Related works

One of the earliest studies about FEE in PD patients was conducted in 2004 by Simons et al. [26] The authors evaluated the capability of 19 PD patients and 25 healthy subjects to pose and imitate different facial expressions. Videos with social interactions were used to evoke emotional responses in the patients faces. The videos were manually analyzed and the participants’ expressiveness was rated according to subjective rating scales, objective facial measurements, and self-questionnaires. The objective measurement was based on the facial action coding system presented in [27], where the facial expression is decomposed according to specific facial muscle movements like rising eyebrows and wrinkling the nose. The results of the study indicated that patients with PD have reduced capability to produce spontaneous facial expressions in all experimental situations. Two years later in [28], the authors presented a work where expressivity and bradykinesia were studied. The authors hypothesized that intentional facial expressions are slowed (bradykinetic) and with less movement in PD patients than in healthy controls. This hypothesis was basically inspired in other intentional movements performed by PD patients, e.g., walking, where bradykinesia is also observed. Digitized videos were evaluated frame-by-frame and the entropy in temporal changes of pixel intensity was measured [29]. The authors found that PD patients had reduced entropy compared to healthy controls, and were significantly slower in reaching a peak expression (*p* < 0.0001), which is directly associated to bradykinesia.

In 2016 Almutiry et al. [30] presented perhaps the only longitudinal study about FEE in PD patients. A total of 8 subjects (4 PD and 4 healthy controls) participated in the study. Patients were recorded for five days per week (once per day) during six weeks while controls were recorded for five days within one week. Participants were requested to produce specific facial expressions while being recorded. The authors used two classical feature extraction methods to localise 27 facial features: Active Appearance Model (AAM) and Constrained Local Model (CLM). The results suggested that PD patients exhibit less movement than controls, which confirms the observations made ten years earlier by Bowers et al. [28].

In 2017, Gunnery et al. [31] studied the coordination of movements across regions of the face in 8 PD patients (4 female). They used the facial action coding system [27, 32] to measure spontaneous facial expressions. The number of activated frames per action unit and their intensity was manually labeled. Correlations were computed for activation values obtained across different regions of the face. The results showed that as severity of facial expression deficit increased, there was a decrease in number, duration, intensity, and co-activation of facial muscle action. In the same year, Bandini et al. [14] classified emotions expressed by 17 PD patients (13 male) and equal number of healthy controls (6 male). Different emotions were evaluated including happiness, anger, disgust, and sadness. Different areas of the face were modeled with 49 landmarks [33, 34], including: eyes, eyebrows, mouth, and nose. A total of 20 features were extracted to define a linear combination of specific reference points. Acted and imitated facial expressions were considered. An support vector machine (SVM) was trained to automatically detect different emotions expressed by participants. The results with imitated expressions showed higher accuracies for healthy controls in most of the emotions. The only case where the PD patients displayed an expression better than the healthy subjects was sadness. When acted expressions were evaluated, the authors found also higher accuracies for healthy subjects than for PD patients.

Other contributions in the topic of FEE in PD include the study of Kang et al [35]. The authors evaluated whether deficiencies in the orofacial movements of PD patients occur in spontaneous and voluntary expressions. Muscular activation (related with specific regions in the face) were studied considering electro-myography signals. Data from the East Asian Dynamic Facial Expression Stimuli (EADFES) database was used [36]. A group with 20 PD patients and 20 healthy controls was evaluated; the authors report limitations of patients to express emotions spontaneously, although the observed dynamics in the movement of the face are similar across all subjects. The study also highlighted the deterioration in the patient’s quality of life due to the presence of “masked face”, affecting social and psychological aspects and increasing their risk to develop depression-related symptoms. The study presented in [15] suggested that PD patients present a deficit in emotion expressivity. According to the results obtained in [15], the deficit seems to be greater for the basic negative emotions (sadness and anger). The basic negative emotions are associated with the following Face Action Units: sadness: 1, 4, 6, 11, 15, 17 and anger: 4, 5, 7, 10, 17, 22-26.

More recently, in another line of work, Grammatikopoulou et al. [37] analyzed facial expressions from images captured with smartphones. Geometric features of the face were extracted and stored in the cloud. A total of 34 participants were recruited, 23 with PD and 11 healthy controls. Patients were divided into three groups according to the facial expression score of the MDS-UPDRS-III scale. The authors extracted two feature sets: one by using the Google Face API and the other one using the Microsoft Face API [38]. The feature sets were composed by reference points on the faces, then two linear regression models were developed (one per feature set) to estimate two different values of the Hypomimia Severity index, namely HSi1 and HSi2. These two indexes were used to classify between Parkinson’s patients and healthy people. The reported sensitivity and specificity values were 0.79 and 0.82, respectively for HSi1 while 0.89 and 0.73 for HSi2. Other contributions include Ali et al. [39]; the authors used OpenFace to evaluate the variance in the action units predictions in PARK dataset [40]. The dataset contains 604 subjects, with 61 PD patients and 534 healthy controls evoking three different expressions. They analyzed three Action Units per expression and an SVM to classify between PD and healthy. The reported accuracies, precision, and recall of 95.6, 95.8, and 94.3, respectively.

In other works, Rajnoha et al. [41] used a face analysis convolutional neural network to extract features over 100 subjects (50 PD and 50 healthy controls) and then used traditional classifiers such as K-nearest neighbors, XGBoost, decision trees, random forest, and SVM to classify PD patients. The reported accuracies for the best classifier was 67.33 for decision trees. Furthermore, Gomez et al. [42] presented a multimodal study based on static and dynamic features for Parkinson’s detection in 4 facial gestures. 17 dynamic features are extracted from a linear combination in an automatic facial mesh [43], and 2048 features are obtained from the maximum peak of the facial gesture. The experiments were carried out on the FacePark-GITA database including 54 participants were recruited, 30 with PD and 24 healthy controls. They reported accuracies of 77.36 and 71.15 only in static and dynamic features respectively, and reported accuracies until 88.76 when both approaches were combined. Additionally, in 2020 Sonawane and Sharma [44] presented a review of automatic techniques and the use of machine learning in detecting emotional facial expressions in PD patients. The authors show that the use of deep learning in this field has not been adequately addressed yet in the classification between healthy people and PD patients. Also, they conducted a pilot experiment based on the use of one CNN from scratch for masked faces detection. In the same year, Jin et al. [45] presented a traditional classifier and recurrent neural networks (RNN) with features based on 106 facial landmarks using Face++. The feature extraction considers the amplitude and tremor of different facial landmarks. The authors evaluated a group with 33 PD patients and 31 healthy controls; the authors reported precision and recall from 0.93 for traditional classifiers and 0.86 for a Long Term Short-Time (LSTM) classifier. The experiments described in [44, 45] show that deep learning-based models can be helpful for classification. Finally, to provide an overall picture, we present Table 1, which contains several machine learning studies related to Parkinson’s disease.

**Table 1:** Comparison of different state-of-the-art machine learning studies on Parkinson’s disease.

Study	PD/HC	Technology	Methods
Simons et al. [26]	19 / 25	Visual analyze	The videos were manually analyzed by experts.
Bowers et al. [28]	12 / 12	Frames difference and Entropy	Entropy calculation over the frame difference between consecutive frames.
Almutiry et al. [30]	4 / 4	Constrained Local Model and Active Appearance Model	27 facial features extracted over 6 week to evaluated variations in expressivity.
Gunnery et al. [31]	8 / -	Visual analyze and correlations	18 AU intensity manually labeled are correlated with different face regions.
Bandini et al. [14]	17 / 17	IntraFace, Linear combination, SVM	20 features defined as the linear combination of 49 facial landmarks are used for expression classification
Kang et al. [35]	20 / 20	Statistical analysis.	Statistical analysis in Electro-myography signals over different faces regions.
Grammatikopoulou et al. [37]	23 / 11	Google Face API, Microsoft Face API, Regressors	27 facial landmarks and 8 facial landmarks were used to calculate a severity index regressor.
Ali et al. [39]	61 / 543	OpenFace, SVM	9 Action Units variance were calculated, and used in PD classification
Rajnoha et al. [41]	50 / 50	Histogram of oriented gradients, Facenet, K-nearest neighbors, XGBoost, decision trees, random forest and SVM.	128 features from Facenet is used in PD classification.
Gomez et al. [42]	30 / 24	MediaPipe, Distance metrics, VGGFace2.	Static and dynamic features from videos were used in a multimodal PD classification.
Sonawane and Sharma. [44]	23 / 30	Convolutional neural network from scratch	A convolutional neural network trained from scratch was used for PD classification.
Jin et al. [45]	33 / 31	Face++, logistic regression, decision trees, random forest, SVM, RNN, LSTM	The amplitude and jitter were calculated on 106 facial landmarks to be used in PD classification.
Our work	30/ 24	CNN, ResNet50, VGGFace2, EmotioNet, Triplet loss, SVM	The amplitude and jitter were calculated on 106 facial landmarks to be used in PD classification.

### Contributions of this work

As shown in the literature review, there is a lack of work in the field of FEE for modeling hypomimia in PD patients with latest affective models including deep learning techniques. One of the main reasons for this lack of deep approaches is the absence of large scale databases with PD patients. In contrast, Face Analysis and Affective Computing research communities have made great efforts to release databases with millions of samples. In this work, we propose to use facial expression analysis and Action Unit domains to improve the PD detection. We propose different domain adaptation techniques [46, 47] to exploit the latest developments in Face Analysis and Face Action Unit (FAU) detection [48]. The main contributions of this paper are: (1) a novel framework to exploit deep face architectures to model hypomimia in PD patients; (2) the comparison of PD detection accuracies based on single images vs. image sequences while the patients elicited various face expressions; (3) we explored different domain adaptation techniques to exploit existing models initially trained either for Face Analysis or to detect FAUs for the automatic discrimination between PD patients and healthy subjects; and (4) a new approach to use triplet-loss learning to improve hypomimia modeling and PD detection.

## Materials and methods

Let’s assume that **w**_FA_ is a model trained for Face Analysis tasks and the representation **x**_FA_ is a feature vector generated by the model (typically from the last layers of a Convolutional Neural Network) from an input face image. This representation **x**_FA_ is learned to describe the face image in a projected space where faces from the same person remain closer than faces from different persons. Similarly, models and representations can be trained for different tasks such as Action Unit recognition (**w**_AU_) (e.g., in the form of facial gestures) or Parkinson’s Disease detection (**w**_PD_). Domain adaptation refers to methods that serve to adapt a representation **x**_A_ trained for the domain A to a new domain B (typically a domain with similar characteristics to A but less information to train). The resulting representation **x**_B_, adapted from **x**_A_, is expected to perform better than a representation trained from scratch for the domain B.

**Fig 1:**
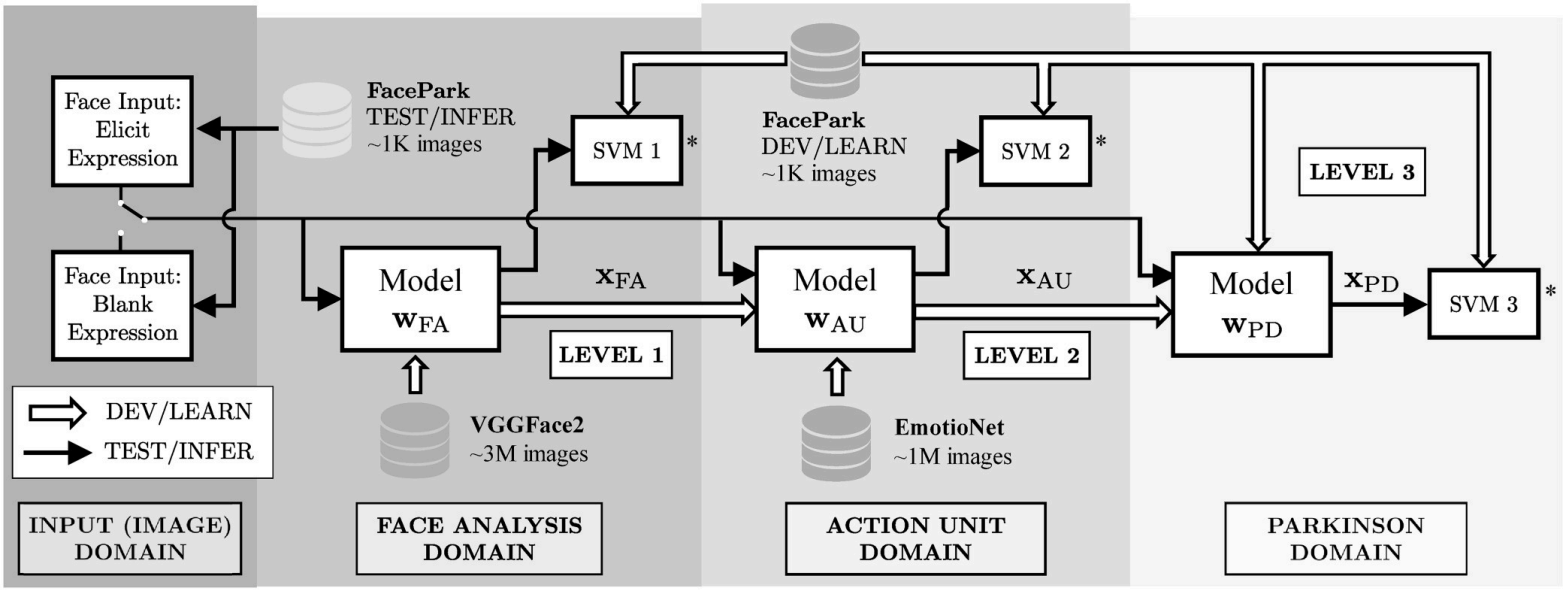
Experimental framework proposed for the development of this work. * SVM 1, SVM 2, and SVM 3 classify between PD and Healthy Control (HC).

We propose an experimental framework where Action Unit features are explored at different levels (or domains). The list of domains and the corresponding underlying hypotheses to be explored are presented below. (See also Fig 1).

**Face Analysis Domain (Level 1)**. We propose to use pre-trained Face Analysis models to extract face representations (namely **x**_FA_) for Parkinson’s Detection.

- *Hypothesis (H1)*: elicited responses intensify the features necessary to model hypomimia in Parkinson’s patients. The representation **x**_FA_ can be improved by incorporating different facial gestures during the acquisition protocol.
- *Experiment*: we evaluate the performance of PD detection for different sequences of face gestures, including right eye wink, left eye wink, smile, anger and surprise, using pre-trained Face Analysis models (**w**_FA_ trained with VGGFace2 [49]).

**Action Unit Domain (Level 2)**. We propose to improve the learned Face Analysis representations (**x**_FA_) for Parkinson Detection by incorporating an Action Unit domain adaptation **w**_AU_ training process:

- *Hypothesis (H2)*: automatic detection of hypomimia is improved when features from the action unit domain are incorporated to the representations. The representation **x**_AU_ performs better for Parkinson Detection than the representation **x**_FA_.
- *Experiment*: the pre-trained models (**w**_FA_) are adapted to the Action Unit domain (**w**_AU_) using the EmotioNet database [50]. Both, the performance of **x**_FA_ and **x**_AU_ are evaluated for Parkinson Detection.

**Parkinson Domain (Level 3)**. We evaluate the performance obtained by representations **x**_PD_ trained with Healthy and Parkinson patients and the Triplet Loss function:

- *Hypothesis (H3)*: similarity learning functions designed to enhance the Parkinson features can serve to improve the capability to detect hypomimia.
- *Experiment*: the Action Unit model (**w**_AU_) is adapted to the Parkinson domain using the Triplet Loss function and the FacePark-GITA database (see Parkinson Domain: FacePark-GITA Section for details).

Details of the methods implemented to validate all hypotheses are presented in Methods.

### Databases

Three different databases are considered in this work. VGGFace2 [49] and EmotioNet [50] which are popular for Face Analysis and Face Action Unit detection, respectively. The third one is a new database composed by PD patients and healthy subjects. It contains face videos of patients suffering from Parkinson’s disease and age-matched healthy controls. This new corpus is called FacePark-GITA. Details of each database are presented below.

#### Face analysis domain: VGGFace2

This database comprises more than 3.31 million faces from 9,131 different subjects. An average of 362.6 images per subject are included [49]. The images were downloaded from Google Image Search. The corpus has large variations in pose, age, lighting, ethnicity, and profession. This database is popular in the Face Recognition community and it has been extensively used to train competitive recognition models [51, 52].

#### Action unit domain: EmotioNet

This database was originally introduced by researchers from the Ohio State University who released the *EmotioNet Challenge* in 2017 [50]. This database contains one million facial expression images collected from the Internet. A total of 950,000 images were annotated by the automatic Action Unit (AU) detection model presented in [50], and the remaining 50,000 images were manually annotated by experts. A total of 12 AUs are included in the corpus.

#### Parkinson domain: FacePark-GITA

The database was created by GITA Lab. The recording of patients is still ongoing and the most updated version of the corpus contains video recordings of 24 healthy participants and 30 PD patients. The videos were recorded at 15 frames per second in non-controlled environment conditions, i.e., light conditions and the background were not controlled prior the recording and differ among participants. PD patients were diagnosed by a neurologist expert and were evaluated according to the MDS-UPDRS-III scale and the Hoehn and Yahr scale (H&Y) [53]. A summary of the clinical and demographic information is presented in Table 2.

**Table 2:** Demographic and clinical information of the participants included in the FacePark-GITA database.

	PD patients		Healthy participants	
	Men	Women	Men	Women
# of Participants	18	12	12	12
Age [years]	70.2 ± 10.4	67.4 ± 10.9	65.3 ± 8.7	65.2 ± 10.1
Age range [years]	52–90	53–87	49–83	49–80
*t* [years]	8.7 ± 5.4	15.6 ± 17.3	—	—
*t* range [years]	2–20	1–45	—	—
MDS-UPDRS-III	35.4 ± 13.9	29.7 ± 12.3	—	—
MDS-UPDRS-III range	16–65	15–54	—	—
H&Y	2.3 ± 0.5	2.5 ± 0.5	—	—
H&Y range	2–3	2–3	—	—

MDS-UPDRS: Movement Disorder Society—Unified Parkinson’s Disease Rating Scale. H&Y: Hoehn & Yahr scale. *t*: Years since diagnosis

The participants of this study were asked to elicit different facial expressions while being recorded. A total of five video-task recordings are included per participant: right eye wink, left eye wink, smile, anger, and surprise. The average duration of each video is 6 seconds. Patients have an average age of 69 years old and healthy subjects were chosen with a similar range of age. Possible bias introduced by age or gender were discarded via a chi-square statistical test (*p* = 0.44) and a Welch’s t-test (*p* = 0.15), respectively.

#### Ethical approval

All of the signals considered in this work were collected in compliance with the Helsinki Declaration and the procedure was approved by the Ethics Committee (CBE-SIU) at the University of Antioquia in Medellín, Colombia. # 19-63-673 of April 25th, 2019. All participants signed a written informed consent before the recording. The individual in this manuscript has given written informed consent (as outlined in PLOS consent form) to publish these case details.

### Methods

#### Image sequences extraction

**Fig 2:**
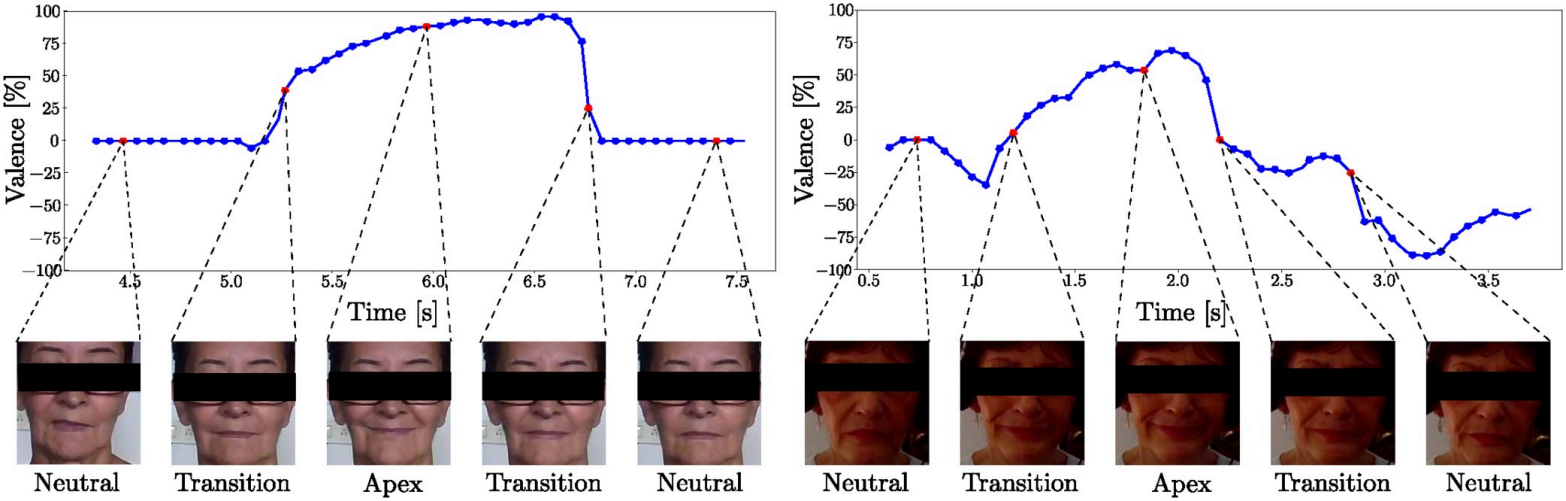
Facial expression stages according to the elicited valence measured with the Affectiva tool. (left) Healthy woman, 63 years old; (right) Woman with Parkinson’s disease, 67 years old, FE item = 2.

Each video from the FacePark-GITA corpus corresponds to a different facial expression: smile, anger, surprise, left eye wink, or right eye wink. Five frames per video-task were extracted with the software Affectiva (available at https://www.affectiva.com/). The curve of valence provided by the software is used as the criterion to select the following sequence of five images/frames per participant on each expression: (i) neutral; (ii) the transition from neutral to the apex (i.e., onset); (iii) apex; (iv) the transition from the apex to neutral (i.e., offset); and (v) neutral. The total of frames used is 1350 frames (5 frames/expression × 5 expressions/user × 54 users). The sequence of images and their direct relation with the valence curve are illustrated in Fig 2. Given the small amount of information provided by individual frames, and considering that extending the analyses to full video-frames would have increased the computational cost and complexity dramatically, we decided to consider multi-frame sequences in a simple information fusion architecture based score fusion [54]. Notice that this approach allows us to capture changes during the production of facial expressions. The general idea was already studied in [55] for speech signals, where the author hypothesised that PD patients have more difficulties to start or stop the movement of muscles and limbs during speech production. The idea was later extended to other motor skills like handwriting and gait [9, 56].

As in the cases of speech, gait, and handwriting, we believe that the same hypothesis holds during the production of facial expressions. Thus, the analysis of multiple-frames in facial expressions should provides useful information to discriminate between PD patients and healthy subjects. The aforementioned idea is implemented by the extraction of the following multi-frame sequences:

- NOnA: Neutral, Onset, and Apex.
- AOffN: Apex, Offset, and Neutral.
- NOnAOffN: Neutral, Onset, Apex, Offset, and Neutral.

#### Face analysis pre-trained model

In this work, we employ the ResNet50 architecture [52], with 50 layers and 25.6M parameters. The architecture adds skip connections to allow gradients to smoothly passed back to early layers. This model is used to generate an initial face representation. The ResNet50 model was originally proposed for general image recognition tasks and later it was retrained with the VGGFace2 database [49] for face recognition. This architecture has been extensively used as a starting point in the Facial expressions analysis [57–59] and Action Units recognition in competitions like Affective Behavior Analysis in-the-wild (ABAW) in FG 2020 [60], ICCV 2021 [61], and CVPR 2022 [62]. The architecture is used as feature extractor by removing the final decision layer. For each face image, the model generates a 1 × 2048 feature vector.

In our experiments we apply Transfer Learning (TL) [63] to adapt from one domain to another (e.g. from Face Analysis to the Action Unit domain). TL are methods where weights from a model originally learned for one task are used as initialization before adjusting the model for a different task. One of the transfer learning techniques consists in freezing intermediate and initial layers to retain their capability to extract general characteristics and retrain the last layers closer to the network output. Re-training of those last layers allows to adapt the original feature space for the new task. These methods are suitable for problems where data is scarce and end-to-end learning approaches fail to find the optimal feature space. The number and size of available databases to model hypomimia in patients suffering from PD are very small (typically less than 100 subjects and less than 1,000 images in total), so we expect that TL techniques will be very useful here to adapt to the Parkinson domain from the Face Analysis domain, where massive datasets are available for learning (millions of images).

#### Face action unit detection models

In addition to the Face Analysis model, in this work we employ two deep neural networks trained from scratch for Face Action Unit (FAU) detection. The architectures employed are based on the popular VGG and ResNet models [48, 64]. The details of the two models are described below:

*VGG-8*: This model contains 8 convolutional layers divided into groups of 2 layers. Each group is followed by a Max pooling layer. Convolutional layers apply a variety of filters to the images and Max-Pooling layers reduce the size of the filtered images. Additionally, dropout is used in the regularization layers to randomly discard neurons in the model and make it less prone to overfitting. The final part of the architecture has a total of six convolutional layers (fully-connected) before the decision layer. The number of neurons per layer is 1024, 512, 256, 128, 64, and 32. The number of parameters of this model is 295,448.

*ResNet-7*: The ResNet model is composed of a total of 7 residual blocks. Each block can be defined as an identity-block or a conv-block. The identity-blocks are the standard blocks used in ResNet, they have a set of convolutional filters and a shortcut connection which bypasses these blocks. This block has the same input and output dimensions. Conv-blocks are the block types where the input and output dimensions do not match. The difference with the identity-block is a convolutional layer in the shortcut to the output. The benefit of these architectures is that in traditional architectures by having a high amount of layers in the training, the problem of error degradation appears. ResNet models with their previous layer shortcut connections are effective in solving this problem [52]. The number of parameters of this model is 366,626.

#### Triplet loss for facial expression analysis

Due to the limited number of samples in the FacePark-GITA database, for the Parkinson domain adaptation we opted for a Triplet Loss learning approach. The Triplet Loss function consists in applying a linear transformation over the data before taking the distance among samples. Given a training data set $S=(x_{i},y_{i})$ with inputs $x_{i}\in R^{d}$ and discrete class labels $y_{i}\in Z$, the goal is to find a transformation to the input data such that reduces the distance between pairs from the same class while increases the distance between pairs from different classes. The Mahalanobis distance defined in Eq 1 is the similarity measure used in this work.

$$\begin{array}{c} d_{M}^{2}\left(x_{i},x_{j}\right)={\left(x_{i}-x_{j}\right)}^{T}M\left(x_{i}-x_{j}\right) \end{array}$$ (1)

where **M** is a positive semi-definite symmetric matrix that can be decomposed as **M** = **T**^*T*^**T**, where **T** denotes a linear transformation matrix. Eq 1 can be rewritten as:

$$\begin{array}{c} d_{M}^{2}\left(x_{i},x_{j}\right)={\left(T\left(x_{i}-x_{j}\right)\right)}^{T}T(x_{i}-x_{j}) \end{array}$$ (2)

$$\begin{array}{cc} & ={{\left\|T(x_{i}-x_{j})\right\|}_{2}^{2}=\|{x_{i}}^{\prime }-{x_{j}}^{\prime }\|}_{2}^{2} \end{array}$$ (3)

The linear transformation **T** can be generalized as Φ(**x**_*i*_), where Φ indicates a kernel function. The resulting distance metric is as follows:

$$\begin{array}{c} d_{M}^{2}\left(x_{i},x_{j}\right)={\left\|\Phi (x_{i})-\Phi (x_{j})\right\|}_{2}^{2} \end{array}$$ (4)

The process to determine the transformed vector Φ(**x**), requires to find a transformation that makes the intra-class distance smaller than the inter-class distance. The general rule which is applied over the data set consists in the following triplets $S_{T}$:

$$\begin{array}{c} S_{T}=\left\{\left(x^{a},y^{a}\right),\left(x^{n},y^{n}\right),\left(x^{p},y^{p}\right)|y^{a}=y^{p},y^{a}\neq y^{n}\right\} \end{array}$$ (5)

where *a*, *p* are samples belonging to the same class, and *n* is a sample from a different class. In our Parkinson detection experiments, the number of classes is two (Healthy and Parkinson). However, we propose to introduce an additional restriction in the triplet. In our experiments, *a*, *p* belong to the same class, but present different face expression. The generation of the triplet $S_{T}$ can be seen as a data augmentation technique. The high number of possible combinations of three elements in a dataset enriches the training process, especially when low number of samples are available. The triplet loss function to be minimized is defined as:

$$\begin{array}{c} L=\sum_{S_{T}}{\left[d_{M}^{2}\left(x^{a},x^{p}\right)-d_{M}^{2}\left(x^{a},x^{n}\right)+\alpha \right]}_{+} \end{array}$$ (6)

where [*z*]_+_ = max(*z*, 0), and *α* ≥ 0 is the minimum margin required between classes.

### Classification and parameter optimization

The automatic classification between healthy people and PD patients is performed using Support Vector Machines (SVMs). The classification of patients with different degree of impairment is performed using SVMs optimized in a one vs. all strategy. In the binary classification experiments with SVMs, linear and Gaussian kernels are considered. The optimization of hyper-parameters is performed in a search grid up to powers of ten with *C* ∈ {10^−4^, 10^−3^, 10^−2^, …, 10^2^, 10^3^} and *γ* ∈ {10^−4^, 10^−3^, 10^−2^, …, 10^3^} for the Gaussian kernel, and for the linear kernel the search considered the same grid to *C* parameter.

All models presented in this work are optimized following a nested 5-folds subject-independent cross-validation strategy and a data augmentation technique with random rotations between -10 and +10 degrees. Each fold has 864, 216, and 270 samples for training, validation, and testing. Classification results are reported in terms of accuracy (Acc), sensitivity (Sens), specificity (Spec), F1-Score (F1), and Area Under the receiver operating characteristic Curve (AUC). In all of the cases, results include values of the optimal hyper-parameters which are found as the mode along the parameters considered along the test folds of each experiment.

## Experiments and results

### Experiment 1: Face analysis domain

#### PD detection based on single face images—Baseline

Individual frames corresponding to each valence level shown in Fig 2 are considered as the baseline to evaluate whether specific frames provide relevant information to discriminate between PD patients and healthy subjects. Feature vectors are obtained from the last layer of the ResNet50 model (see Section Face Analysis pre-trained model). Table 3 summarizes the results.

**Table 3:** Results of classification using a single image from the extracted image sequence.

E.S.	Kernel*	Acc[%]	Sens[%]	Spec[%]	F1[%]
Neutral	*C*=1e+01; *γ*=1e-04	69.0 ± 10.1	74.0 ± 11.6	63.0 ± 9.7	67.8 ± 10.1
Apex	*C*=1e+01; *γ*=1e-04	70.0 ± 9.1	84.4 ± 7.9	53.3 ± 24.0	61.0 ± 18.6
Onset	*C*=1e+01; *γ*=1e-04	71.4 ± 3.2	88.6 ± 7.0	50.0 ± 9.0	63.1 ± 6.6
Offset	*C*=1e+01; *γ*=1e-04	71.6 ± 5.2	79.5 ± 3.3	61.9 ± 13.5	68.6 ± 8.2
Neutral	*C*=1e-03	70.8 ± 9.6	77.3 ± 10.2	63.0 ± 9.7	69.3 ± 9.7
Apex	*C*=1e-03	70.8 ± 9.1	83.7 ± 7.3	55.7 ± 21.6	63.8 ± 16.3
**Onset**	***C*=1e-02**	**72.9 ± 4.2**	**88.6 ± 7.8**	**53.4 ± 7.7**	**66.1 ± 5.9**
Offset	*C*=1e-01	72.8 ± 4.3	81.5 ± 4.5	61.9 ± 13.5	69.2 ± 7.9

**E.S**.: Expression stage. First four rows: Gaussian kernel. Last four rows: Linear kernel.

*Column with optimal hyper-parameters.

Note that there is almost no difference among the accuracies obtained with the frames of each expression stage. Perhaps the only thing to highlight is the high sensitivity (88.6%) of the Onset stage, which likely indicates that this stage is maybe a good choice to model hypomimia in specific frames within a video. This preliminary observation will be further elaborated in the next experiments.

#### PD detection based on image sequences

The three image sequences introduced in Image sequences extraction are used here to discriminate between PD patients and Healthy Control (HC) subjects. Table 4 shows the results obtained when the changes in the production of facial expressions are incorporated by feature vectors extracted from multi-frame sequences.

**Table 4:** Results of the classification using different combinations of the extracted frames sequences.

Sequences	Kernel*	Acc[%]	Sens[%]	Spec[%]	F1[%]
NOnA	*C*=1e+02; *γ*=1e-04	77.4 ± 8.7	89.3 ± 4.6	63.0 ± 16.1	72.9 ± 11.2
AOffN	*C*=1e+01; *γ*=1e-04	76.3 ± 8.0	86.8 ± 12.0	63.5 ± 22.4	69.2 ± 17.8
NOnAOffN	*C*=1e+01; *γ*=1e-04	77.2 ± 8.6	86.1 ± 14.8	67.2 ± 10.3	74.2 ± 8.5
NOnA	*C*=1e-03	78.2 ± 9.8	90.1 ± 5.2	63.8 ± 17.1	73.8 ± 12.6
AOffN	*C*=1e-03	77.8 ± 9.1	88.8 ± 9.4	64.2 ± 24.1	70.4 ± 20.5
**NOnAOffN**	***C*=1e-03**	**78.4 ± 7.1**	**87.8 ± 11.4**	**67.7 ± 11.6**	**75.4 ± 7.9**

First three rows: Gaussian kernel. Last three rows: Linear kernel.

*Column with optimal hyper-parameters.

The results obtained by the multi-frame sequences are better than those obtained with individual frames. The improvement is around 7% and the best result is obtained with the two cases where the sequence NOnA is included, which is focused on modeling information in the transition between neutral and the production of a certain expression. It is also worth to highlight that sensitivity is near 90% in all of the cases, while specificity is rather low (around 64%). This indicates that the proposed approach is good to detect patients but not as good to detect healthy controls.

This result validates the hypothesis *H1* about the existence of useful information related to hypomimia in the elicited facial expressions. Given this clear improvement, the next experiments will include only feature vectors extracted from multi-frame sequences.

### Experiment 2: Action unit domain

**Fig 3:**
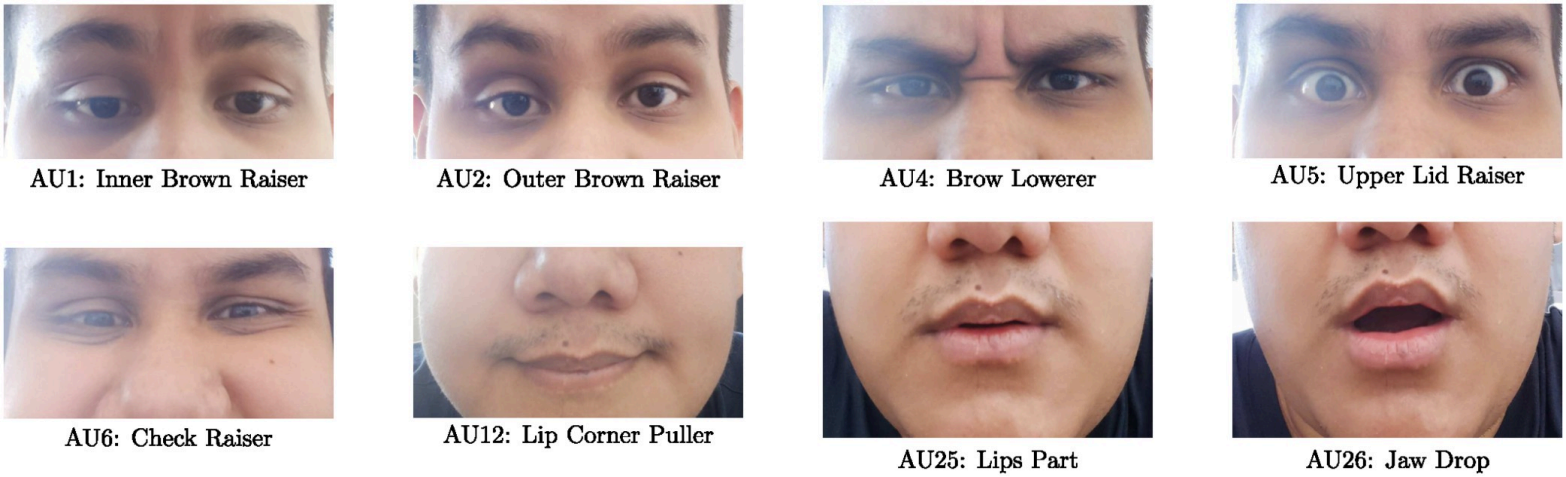
Action Units defined for the Experiment 2.

This experiment intends to incorporate information from the Action Unit domain to improve Parkinson’s Disease (PD) detection. In this case the EmotioNet database is used to create an appropriate facial representation space. The first step consists in selecting AUs that provide suitable information to perform the automatic classification between PD patients and healthy subjects. We selected a subset of AUs according to [32] adequate for the facial expressions included in the recording tasks of the FacePark-GITA database. We included the AUs 1, 2, 4, 5, 6, 12, 25, and 26 from EmotioNet dataset; Motivated by the fact that AUs 4,5,25, and 26 are related to the anger expression (negative expression), the AUs 6, 12, and 25 are related to smile expression (positive expression), and the AUs 1, 2, 5, 25, and 26 are related to surprise (others expression) [65]. Fig 3 shows the set of selected AUs.

#### Adaptation from face analysis models

The process to adapt the convolutional models from one domain to another consists in freezing different percentages of the layers and retraining the remaining portion. The data with the selected AUs from the EmotioNet dataset are used here to retrain the models. In this case we evaluate three percentages of layers frozen during the retraining of the ResNet50 model (originally trained for Face Analysis): freezing 50% (Freeze 50—20.5M trainable parameters), freezing 75% (Freeze 75—16.0M trainable parameters), and freezing 100%. Note that the freezing 100% model is taken as the Baseline and corresponds to the case where no action units information is incorporated (**x**_FA_). After the convolutional layers, a fully connected layer is added for the classification of the 8 selected AUs (see Fig 3). The result of the retraining process and its performance to classify the AUs is shown in Table 5 in terms of AUC and EER values. The accuracy varies depending of the FAU and the percentage of layers frozen. The FAUs numbers 6, 12, and 25 reached accuracies around 90%, while the rest of the FAUs achieved performances around 80%.

**Table 5:** FAU detection results of the VGGFace2 model after retraining with the EmotioNet database.

Models	Metrics	AU 1	AU 2	AU 4	AU 5	AU 6	AU 12	AU 25	AU 26
Baseline (**x**_FA_)	AUC	0.83	0.83	0.87	0.80	0.94	0.95	0.92	0.80
	EER [%]	24.58	23.78	21.01	27.13	12.82	12.11	15.38	27.32
Freeze 75 (**x**_AU_)	AUC	0.84	0.84	0.86	0.84	0.92	0.93	0.95	0.85
	EER [%]	21.84	20.80	19.90	21.65	14.34	10.42	8.63	22.48
Freeze 50 (**x**_AU_)	AUC	0.84	0.87	0.87	0.87	0.93	0.95	0.90	0.83
	EER [%]	20.56	19.29	18.92	19.53	13.22	10.58	10.99	24.32

The representations **x**_AU_ obtained by the retrained models are further used to classify between PD patients and healthy subjects of the FacePark-GITA corpus. The results obtained with the Freeze 75 and Freeze 50 models are shown in Tables 6 and 7, respectively. The results for the Baseline model correspond to those previously shown in Table 4. Optimal hyper-parameters found in the 5-fold cross-validation process are also included in every experiment.

**Table 6:** PD classification results using the Freeze 75 model.

Sequence	Kernel*	Acc[%]	Sens[%]	Spec[%]	F1[%]
NOnA	*C*=1e+01; *γ*=1e-04	84.2 ± 5.4	90.0 ± 8.3	77.2 ± 10.8	82.3 ± 6.3
AOffN	*C*=1e+02; *γ*=1e-04	81.6 ± 8.6	87.8 ± 7.4	73.9 ± 11.5	80.0 ± 9.5
NOnAOffN	*C*=1e+02; *γ*=1e-04	86.7 ± 8.9	91.2 ± 4.7	81.6 ± 15.5	85.5 ± 10.2
NOnA	*C*=1e-01	84.7 ± 5.4	89.5 ± 9.4	78.9 ± 11.3	82.9 ± 6.5
AOffN	*C*=1e-01	82.6 ± 9.6	87.8 ± 8.3	76.1 ± 13.3	81.2 ± 10.4
**NOnAOffN**	***C*=1e-01**	**87.3 ± 8.0**	**90.6 ± 5.0**	**83.6 ± 13.1**	**86.6 ± 8.8**

First three rows: Gaussian kernel. Last three rows: Linear kernel.

*Column with optimal hyper-parameters.

**Table 7:** PD classification results using the Freeze 50 model.

Sequence	Kernel*	Acc[%]	Sens[%]	Spec[%]	F1[%]
**NOnA**	***C*=1e+01; *γ*=1e-04**	**83.1 ± 6.0**	**87.7 ± 12.4**	**77.5 ± 10.2**	**81.1 ± 6.5**
AOffN	*C*=1e+01; *γ*=1e-04	81.3 ± 7.5	86.3 ± 13.0	75.6 ± 3.6	80.1 ± 6.8
NOnAOffN	*C*=1e+00; *γ*=1e-04	81.9 ± 9.2	97.4 ± 2.5	63.4 ± 17.7	75.5 ± 14.3
NOnA	*C*=1e-01	82.1 ± 6.8	85.0 ± 13.8	78.6 ± 11.0	80.2 ± 7.7
AOffN	*C*=1e-01	80.0 ± 7.6	83.4 ± 12.7	76.1 ± 4.4	79.1 ± 7.2
NOnAOffN	*C*=1e-01	80.2 ± 11.1	84.3 ± 8.5	75.3 ± 19.1	78.3 ± 13.0

First three rows: Gaussian kernel. Last three rows: Linear kernel.

*Column with optimal hyper-parameters.

**Fig 4:**
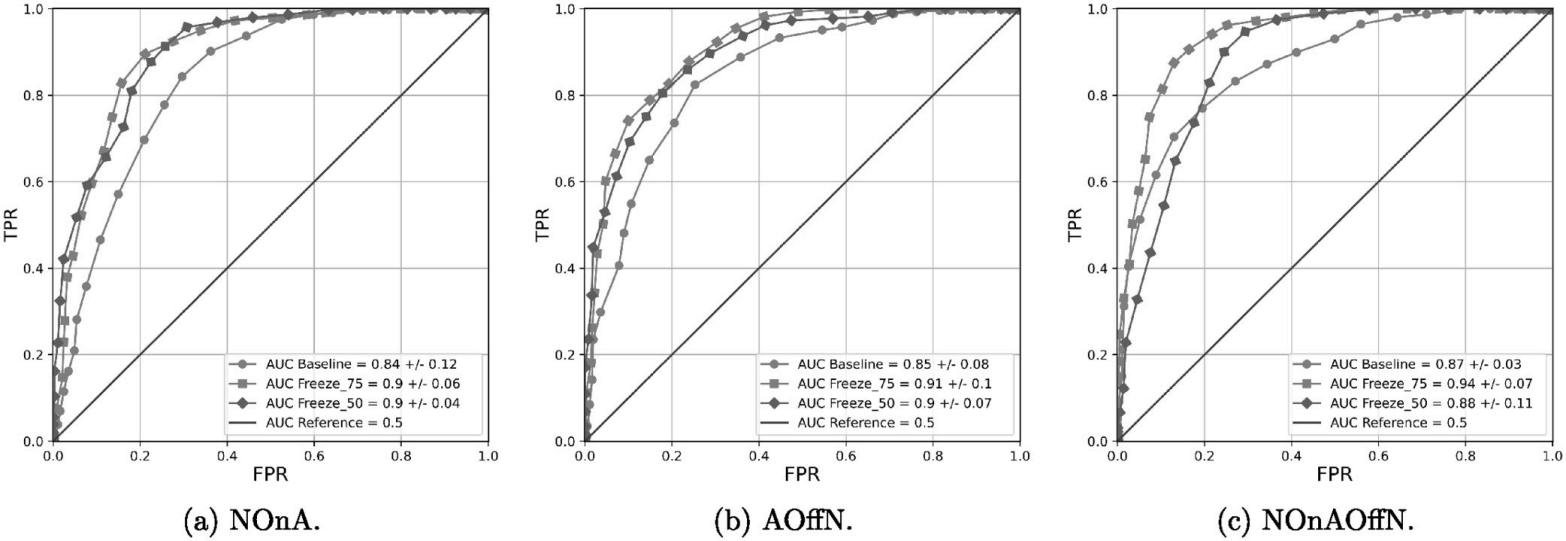
PD classification ROC curves obtained from the different input sequences in the retrained Freeze models.

Note that the Freeze 75 exhibits higher accuracies than the Freeze 50, indicating that considerable information from the Face Analysis domain is still useful to obtain good results in the classification between PD patients and healthy subjects. More interestingly, note that the best accuracy obtained with the Freeze 75 model in Table 6 (87.3%) is 8.9% higher than the best result obtained when only a Face Analysis model is considered (Table 4). This result supports our second hypothesis (*H2*), the idea of incorporating information from the Action Unit domain to the Face Analysis domain to improve detection of hypomimia in PD patients. The benefits of including information of the Action Unit domain are also shown in Fig 4, where the ROC curves obtained with the Freeze 75, Freeze 50, and Baseline models are presented.

Note that the models used until this point of the study are based on architectures originally trained for Face Analysis tasks (ResNet50). Now we want to evaluate the importance of this initialization based on a Face Analysis training processes.

#### Training action unit models from scratch

The previous scenario studied the performance of pre-trained models with high number of parameters learned from the Face Analysis domain after adaptation to the Action Unit domain. In this section we will train FAU detection models from scratch. ResNet50 requires to optimize more than 20M parameters. Conversely, the VGG-8 and ResNet-7 architectures proposed in Face Action Unit detection models Section require the optimization of 295,448 and 366,626 parameters respectively. These reduced architectures are trained with the same data as those considered previously to retrain the Freeze 50 and Freeze 75 models. Table 8 shows the results with the AUC values obtained when the different AUs are detected. Note that these results are higher than those reported in Table 5 where greater number of parameters are optimized. However, the ResNet50 was originally trained for face recognition tasks, where face gestures are features to be excluded from the representation space. This result indicates that a simpler model might provide high enough AUs discrimination performance to be used in the classification between PD patients and healthy controls.

**Table 8:** FAU detection results of the VGG-8 and ResNet-7 training with EmotioNet database.

Models	Metrics	AU 1	AU 2	AU 4	AU 5	AU 6	AU 12	AU 25	AU 26
**ResNet-7**	AUC	0.92	0.93	0.91	0.91	0.96	0.97	0.97	0.91
	EER [%]	15.25	14.21	16.20	13.58	10.05	8.42	7.39	16.32
**VGG-8**	AUC	0.89	0.87	0.89	0.90	0.96	0.96	0.96	0.90
	EER [%]	16.59	16.08	16.88	14.87	9.51	8.11	7.83	16.55

Tables 9 and 10 show the results obtained when the aforementioned models, created with the reduced architectures, are used to discriminate between PD patients and healthy subjects. Note that no additional training is performed with data from Parkinson’s disease patients. The best results are obtained when the ResNet-7 architecture is considered with features extracted from the NOnAOffN sequence.

**Table 9:** PD classification results using the VGG-8 model.

Sequence	Kernel*	Acc[%]	Sens[%]	Spec[%]	F1[%]
NOnA	*C*=1e+01; *γ*=1e-02	58.3 ± 3.7	94.6 ± 4.8	14.1 ± 6.3	24.0 ± 9.8
AOffN	*C*=1e+01; *γ*=1e-03	65.6 ± 8.6	80.6 ± 8.0	47.6 ± 16.4	58.1 ± 12.9
NOnAOffN	*C*=1e+01; *γ*=1e-04	62.7 ± 8.3	66.4 ± 10.0	58.2 ± 13.1	60.9 ± 8.6
NOnA	*C*=1e-02	67.4 ± 8.3	72.4 ± 9.4	61.3 ± 9.8	66.0 ± 8.2
**AOffN**	***C*=1e-02**	**67.6 ± 5.8**	**70.6 ± 7.4**	**63.9 ± 13.5**	**65.9 ± 7.3**
NOnAOffN	*C*=1e-02	64.9 ± 7.7	71.0 ± 4.5	57.7 ± 16.1	62.2 ± 11.0

First three rows: Gaussian kernel. Last three rows: Linear kernel.

*Column with optimal hyper-parameters.

**Table 10:** PD classification results using the ResNet-7 model.

Sequence	Kernel*	Acc[%]	Sens[%]	Spec[%]	F1[%]
NOnA	*C*=1e+03; *γ*=1e-04	73.0 ± 9.5	75.9 ± 18.7	69.7 ± 17.8	68.9 ± 12.3
AOffN	*C*=1e+01; *γ*=1e-02	73.4 ± 9.9	81.7 ± 15.6	63.6 ± 8.9	70.5 ± 9.5
**NOnAOffN**	***C*=1e+03; *γ*=1e-04**	**78.8 ± 6.4**	**79.3 ± 9.8**	**78.2 ± 12.8**	**77.6 ± 6.7**
NOnA	*C*=1e-02	74.1 ± 6.9	82.2 ± 19.4	64.5 ± 11.4	69.3 ± 6.1
AOffN	*C*=1e-02	72.4 ± 10.8	84.2 ± 16.5	58.2 ± 8.6	68.1 ± 9.6
NOnAOffN	*C*=1e-01	78.3 ± 7.3	80.1 ± 10.6	76.2 ± 10.1	77.3 ± 7.4

First three rows: Gaussian kernel. Last three rows: Linear kernel.

Column with optimal hyper-parameters.

Although 78.3% could be considered a good accuracy, it is still far from the best result obtained with the ResNet50 Freeze 75 model (87.3% in Table 6), indicating that the FAU domain is missing certain features present in the Face Analysis domain.

**Fig 5:**
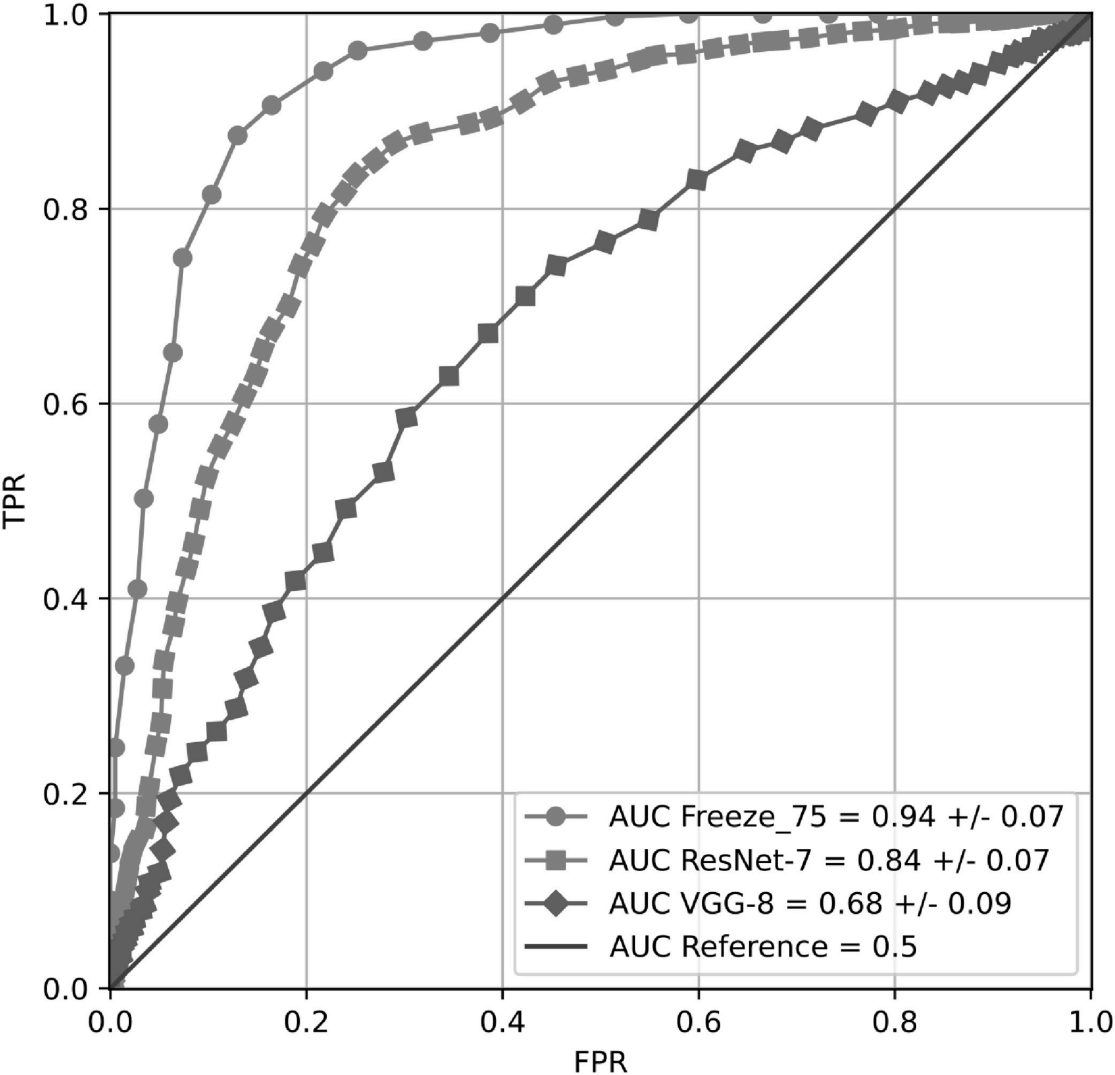
Comparison between PD classification ROC curves obtained using the NOnAOffN sequence in the Freeze 75, ResNet-7 and VGG-8.

Fig 5 shows three ROC curves where results with Freeze 75, ResNet-7, and VGG-8 are compared. The superiority of the Freeze 75 model is clearly observed, supporting the advantages of initializing the models using the Face Analysis domain.

### Experiment 3: Parkinson’s domain (PD detection)

In this section, different strategies are explored with the aim to evaluate their suitability to model specific patterns that appear on the face of PD patients. First, a simple model based on CNNs is trained from scratch and later the use of a triplet loss function is explored to evaluate whether the classification performance of PD patients vs. Healthy Control (HC) subjects is improved. The triplet loss function modifies the original representation space such that the inter-class separability is increased while the intra-class separability is reduced. The modified feature vectors are called *embedded vectors*.

#### Training Parkinson detection models from scratch

The previous section showed the benefits of the Action Units domain adaptation. This experiment is performed considering the NOnAOffN sequence from the FacePark-GITA database as training and test data. The use of this sequence is motivated by the need of maximizing the amount of data in the training process. The models are directly trained, i.e., from scratch, with randomly initialized weights with cross-entropy as the loss function. Table 11 shows the results of this experiment, which can be considered also as a baseline regarding the use of deep learning -based architectures.

**Table 11:** Classification results using a CNN architecture trained from scratch with the NOnAOffN sequences.

Model	Acc[%]	Sens[%]	Spec[%]	F1[%]
VGG-8	67.7 ± 11.5	52.9 ± 32.8	86.0 ± 17.5	55.5 ± 19.8
ResNet-7	71.7 ± 15.4	81.4 ± 19.4	59.7 ± 21.2	66.1 ± 18.8

Table 11 shows the performance of the models created from scratch trained with the NOnAOffN sequences of the FacePark-GITA database. The results show that the accuracy of the VGG-8 with randomly initialized weights are comparable to those obtained when the adapted Action Unit model is considered (approximately 67.7% in both cases). However, it can also be observed that these results have high levels of variance compared to the results in Table 9 (i.e. 32.8% and 7.4% Sens variance for PD domain and AU domain respectively). When comparing results in Tables 10 and 11, it can be observed that ResNet-7 has higher variance but lower accuracy than the adapted Action Unit model (71.7% and 78.8%, respectively). This is likely due to the lack of enough data to appropriately train the model, which highlights the convenience of applying TL techniques.

#### Triplet loss in face analysis models adapted to the action unit domain

The Freeze 75 and Freeze 50 models are trained with the triplet loss function strategy and two new models are obtained, namely Triplet 75 and Triplet 50, respectively. The FacePark-GITA database is divided into a 5-fold partition for the training of each Triplet model and the SVM classifier. The classification results obtained when using the embedded vectors are shown in Table 12 for the Triplet 75 model, and in Table 13 for the Triplet 50 model.

**Table 12:** PD classification results of classification with the Triplet 75 model.

Sequence	Kernel*	Acc[%]	Sens[%]	Spec[%]	F1[%]
NOnA	*C*=1e+01; *γ*=1e-04	85.2 ± 7.4	87.6 ± 5.8	82.5 ± 12.6	84.5 ± 8.2
AOffN	*C*=1e+01; *γ*=1e-04	86.0 ± 6.1	91.4 ± 6.9	79.5 ± 7.1	84.9 ± 6.2
NOnAOffN	*C*=1e+01; *γ*=1e-04	86.0 ± 9.0	92.1 ± 6.9	78.7 ± 13.4	84.5 ± 10.1
NOnA	*C*=1e-01	84.4 ± 6.6	87.4 ± 4.4	80.9 ± 13.3	83.4 ± 7.6
AOffN	*C*=1e-01	85.0 ± 5.9	90.3 ± 6.4	78.7 ± 7.1	84.0 ± 6.1
**NOnAOffN**	***C*=1e-01**	**86.1 ± 9.6**	**91.4 ± 7.5**	**79.9 ± 13.5**	**85.0 ± 10.5**

First three rows: Gaussian kernel. Last three rows: Linear kernel.

*Column with optimal hyper-parameters.

**Table 13:** PD classification results of classification with the Triplet 50 model.

Sequence	Kernel*	Acc[%]	Sens[%]	Spec[%]	F1[%]
NOnA	*C*=1e+01; *γ*=1e-04	78.9 ± 5.5	84.3 ± 10.9	72.4 ± 11.3	76.7 ± 6.1
AOffN	*C*=1e+03; *γ*=1e-04	73.2 ± 8.7	69.1 ± 16.9	78.3 ± 4.0	72.2 ± 8.3
NOnAOffN	*C*=1e+02; *γ*=1e-04	75.8 ± 11.8	77.4 ± 15.5	74.3 ± 16.2	74.2 ± 12.5
**NOnA**	***C*=1e-01**	**80.7 ± 6.6**	**86.4 ± 13.2**	**73.9 ± 11.8**	**78.1 ± 7.4**
AOffN	*C*=1e-01	76.3 ± 8.7	79.1 ± 17.4	73.3 ± 7.4	74.5 ± 8.6
NOnAOffN	*C*=1e-01	77.1 ± 10.2	83.0 ± 10.7	69.9 ± 19.8	73.9 ± 13.2

First three rows: Gaussian kernel. Last three rows: Linear kernel.

*Column with optimal hyper-parameters.

Note that the Triplet 75 model exhibits better accuracy (86.0%) than the Triplet 50 (80.7%). Since the best accuracies in the previous experiments with the Freeze 75 and Freeze 50 models were 87.3% and 83.1%, these new results obtained with the triplet loss strategy likely indicate that the embedding approach does not provide advantages over the use of transfer learning and freezing of layers. This observation is also supported in the fact that the number of parameters to be optimized has not been reduced, so in principle, there is no reason for using the triplet loss function in these two scenarios.

#### Triplet loss in FAU detection trained from scratch

In this experiment the VGG-8 and ResNet-7 models are retrained considering the triplet loss function, creating two new models, namely Triplet-VGG8 and Triplet-ResNet7, respectively. These new models are used to extract embedded vectors for further classification between PD patients and healthy subjects. The results obtained with the Triplet-VGG8 and Triplet-ResNet7 embedded vectors are shown in Tables 14 and 15, respectively.

**Table 14:** PD classification results using the Triplet-VGG8 model.

Sequence	Kernel*	Acc[%]	Sens[%]	Spec[%]	F1[%]
NOnA	*C*=1e+01; *γ*=1e-04	71.2 ± 8.8	76.4 ± 14.0	64.9 ± 12.8	68.7 ± 8.2
AOffN	*C*=1e+03; *γ*=1e-03	69.9 ± 9.6	67.4 ± 8.2	72.9 ± 13.1	69.8 ± 9.6
NOnAOffN	*C*=1e+00; *γ*=1e-03	66.0 ± 8.4	79.0 ± 10.5	50.7 ± 21.0	58.2 ± 14.9
**NOnA**	***C*=1e-02**	**72.7 ± 7.2**	**80.8 ± 13.4**	**62.6 ± 11.5**	**69.1 ± 7.9**
AOffN	*C*=1e+01	70.3 ± 7.0	74.9 ± 9.4	64.8 ± 13.2	68.3 ± 7.8
NOnAOffN	*C*=1e+01	65.3 ± 5.1	65.0 ± 3.9	65.4 ± 13.7	64.1 ± 6.8

First three rows: Gaussian kernel. Last three rows: Linear kernel.

*Column with optimal hyper-parameters.

**Table 15:** PD classification results using the Triplet-ResNet7 model.

Sequence	Kernel*	Acc[%]	Sens[%]	Spec[%]	F1[%]
NOnA	*C*=1e+03; *γ*=1e-04	82.1 ± 8.8	87.2 ± 7.4	76.0 ± 14.3	80.5 ± 10.1
AOffN	*C*=1e+02; *γ*=1e-03	78.2 ± 12.9	79.6 ± 13.6	76.3 ± 16.3	77.3 ± 13.0
NOnAOffN	*C*=1e-01; *γ*=1e-03	69.9 ± 10.8	82.8 ± 15.2	54.7 ± 22.0	61.8 ± 17.9
**NOnA**	***C*=1e-01**	**82.4 ± 8.5**	**89.2 ± 5.9**	**74.1 ± 12.6**	**80.7 ± 9.7**
AOffN	*C*=1e-01	76.2 ± 11.0	78.9 ± 12.5	72.8 ± 12.7	75.3 ± 11.0
NOnAOffN	*C*=1e-02	79.6 ± 5.4	89.0 ± 11.0	68.6 ± 10.3	76.5 ± 5.1

First three rows: Gaussian kernel. Last three rows: Linear kernel.

*Column with optimal hyper-parameters.

Note that there is an improvement in both models compared to those based on VGG-8 and ResNet-7 where the triplet loss function was not applied. In the first case the improvement is around 5.1% (from 67.6% to 72.7%) and in the second case is around 3.6% (from 78.8% to 82.4%). These results partially validates our third hypothesis (*H3*) indicating that loss functions designed to learn from the PD domain serve to improve the performance of PD classification. It is not only interesting to highlight the improvement achieved when using the triplet loss function, but also to note that the best result obtained with the Triplet-ResNet7 model is competitive compared to the best accuracy previously obtained with the Freeze 75 model. Although the accuracy in the second one is 4.9% above the first one, Freeze 75 requires 17,815,520 more parameters to be optimized than Triplet-ResNet7, which might indicate a better generalization capability. Further experiments with additional data are required to validate this hypothesis.

**Fig 6:**
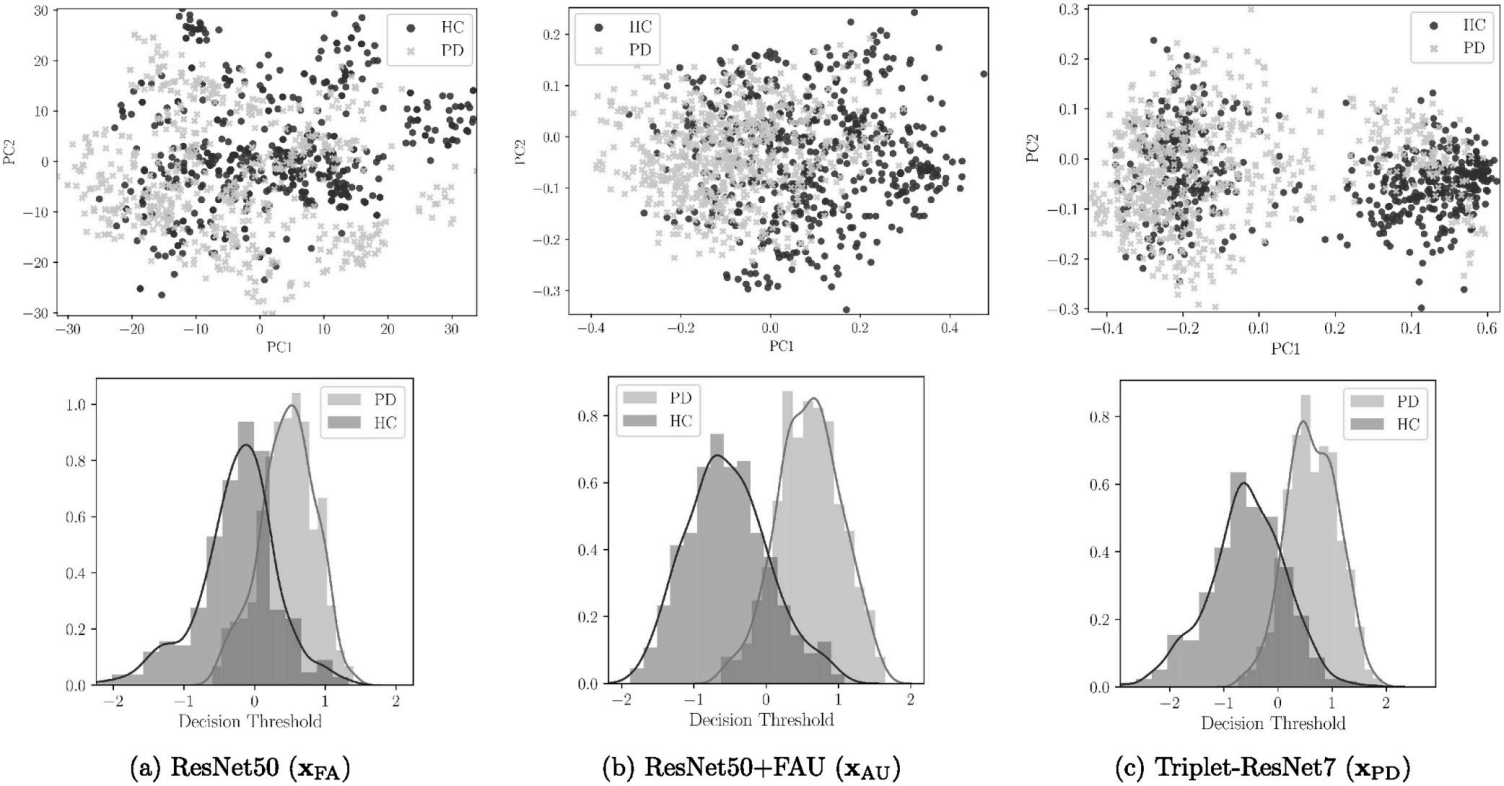
(Up) Principal components spaces generated from the features of the different models and (Bottom) score distributions of PD patients and Healthy Control (HC) subjects obtained by the SVM classifier.

PCA is now used to create a 2D representation of the feature spaces learned in previous experiments. Fig 6 shows the feature spaces and the distribution of the classification scores. The figure shows a superior discrimination capability of the **x**_AU_ feature space (ResNet50 adapted to the FAU domain). The representation obtained by the Triplet-ResNet7 model shows a larger margin between classes but the misclassification errors decrease the performance.

Finally, we performed 25 random nested cross-validations with the hyper-parameters found previously to generate accuracy sets to realize the Kruskal-Wallis test between the three classification models. The Kruskal-Wallis test shows a p-value (5.70e-13 <0.05), demonstrating a difference between the mean of the models’ accuracies. Following, we applied Mann-Whitney U tests for post hoc pairwise comparison of models and a Bonferroni-corrected significance value (*α*_cor_ = 0.05/3 ≈ 1.66e-2). Table 16 shows the results of the Mann-Whitney U test and if we reject the null hypothesis H_0_.

**Table 16:** Pairwise comparison results with Mann-Whitney U test and Bonferroni correction for the classification models.

Models	p-value (Mann-Whitney U test)	H_0_ is rejected (<*α*_cor_≈ 1.66e-2)
ResNet50 vs. Freeze 75	5.96e-13	Yes
ResNet50 vs. Triplet-ResNet7	9.10e-3	Yes
Freeze 75 vs. Triplet-ResNet7	4.73e-8	Yes

## Discussion and conclusion

This study presents a novel approach where deep learning methods are used to model hypomimia in PD patients. Videos with the face of people while eliciting facial expressions are considered for the study. Frames of the recorded videos are segmented into different stages during the production of elicited expressions: neutral, onset-transition, apex, offset-transition, and neutral. This approach exhibits improvements of up to 5.5% in accuracy (from 72.9% to 78.4%) with respect to classical approaches where single frames are considered. These results suggest that dynamics information is more suitable to model hypomimia in PD patients. We are aware of the fact that the presented approach does not completely exploit the video dynamics; however, the incorporation of frames in different stages during the production of facial expressions shows to be a good and computationally affordable approach.

Later, information from the Action Unit domain is incorporated in the model by means of transfer learning methods. Transfer learning was performed considering the complete architecture of a base model previously trained with massive data and then freezing some layers to fine-tune the remaining layers with the smaller action units data. Results freezing 75% and 50% of the layers are reported. The results show that the Action Unit domain adaptation provides an improvement of 8.9%, from 78.4% to 87.3% of accuracy in PD detection. These results confirm that domain adaptation via transfer learning methods is a good strategy to model hypomimia in PD patients. Considering the good results and also the fact that only up to five images per participant are considered in the experiments, we believe that this study is a step forward in the development of automatic methods for the detection and monitoring of PD symptoms related with the production of facial expressions.

With the aim of finding lighter approaches suitable to be used in portable devices, other experiments with reduced architectures like VGG-8 and ResNet7 were also addressed. However, the results were not satisfactory, i.e., the maximal accuracies in these cases were 67.6% and 78.8%, respectively. The results were further improved up to 72.7% and 82.4% when the triplet loss strategy was considered.

**Fig 7:**
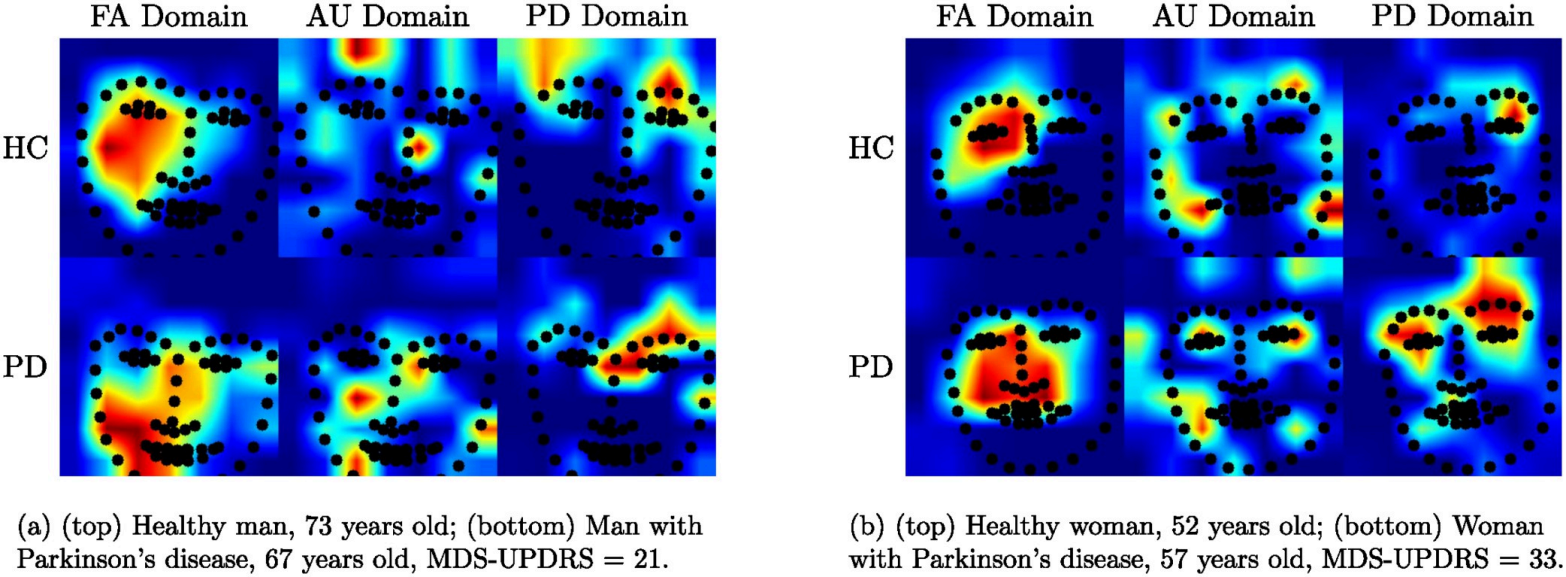
Heatmaps representations in two different tasks over the three domains studied: (a) Right wink task and (b) Smile task.

Fig 7 illustrates the activation maps of the ResNet50 model and the face landmarks through the three domains used in this work (we do not show the face images for privacy reasons). Each row in the figure shows the changes in the activation maps in three different columns: FA Domain which corresponds to the classical Face Analysis domain and focuses broadly on the faces of the participants. The AU domain which shows concentration in more specific regions over the face, where these regions are highly related to the facial action units. Notice that the regions activated in the AU Domain of Fig 7 (second column) are related to the right wink task, while the AU Domain images in Fig 7 show more intensive regions over the lips, which are closely related to the smile task. And finally, the third column which corresponds to the Parkinson’s domain when the Triplet loss function is applied. Notice that in this case the concentration in the upper face area is intensified, indicating that it is the one that provides better discriminative capability to detect PD.

The computer vision approach applied to model hypomimia effects present limitations. More research is needed before these approaches have a direct impact on patients’ lives. The study of connections and patterns between emotions, facial expressions, and hypomimia symptoms will allow to improve computer vision approaches. In this respect, the lack of large dataset acquired by multidisciplinary teams including PD experts and machine learning experts is a major handicap for the advancement of the research community. The availability of larger corpus will allow to study the use of more sophisticated machine learning architectures such as MobileNets, ShuffleNet, Multiresolution ensemble structures or other technologies to integrate information provided by video sequences, including video tracking of facial features and other modalities [54], like speech, gait, handwriting [11], and human-computer interaction signals [66].
