# Correction: Exploring facial expressions and action unit domains for Parkinson detection

**DOI:** 10.1371/journal.pone.0320872

**Published:** 2025-03-20

**Authors:** Luis F. Gomez, Aythami Morales, Julian Fierrez, Juan Rafael Orozco-Arroyave

There is an error in the affiliation of the fourth author. Juan Rafael Orozco-Arroyave is not affiliated with #2 but with #1: Faculty of Engineering, Universidad de Antioquia, Medellín, Antioquia, Colombia.
